# Towards System Calibration of Panoramic Laser Scanners from a Single Station

**DOI:** 10.3390/s17051145

**Published:** 2017-05-17

**Authors:** Tomislav Medić, Christoph Holst, Heiner Kuhlmann

**Affiliations:** Institute of Geodesy and Geoinformation, University of Bonn, Nussallee 17, 53115 Bonn, Germany; c.holst@igg.uni-bonn.de (C.H.); heiner.kuhlmann@uni-bonn.de (H.K.)

**Keywords:** panoramic terrestrial laser scanners, system calibration, self-calibration, mechanical calibration parameters

## Abstract

Terrestrial laser scanner measurements suffer from systematic errors due to internal misalignments. The magnitude of the resulting errors in the point cloud in many cases exceeds the magnitude of random errors. Hence, the task of calibrating a laser scanner is important for applications with high accuracy demands. This paper primarily addresses the case of panoramic terrestrial laser scanners. Herein, it is proven that most of the calibration parameters can be estimated from a single scanner station without a need for any reference information. This hypothesis is confirmed through an empirical experiment, which was conducted in a large machine hall using a Leica Scan Station P20 panoramic laser scanner. The calibration approach is based on the widely used target-based self-calibration approach, with small modifications. A new angular parameterization is used in order to implicitly introduce measurements in two faces of the instrument and for the implementation of calibration parameters describing genuine mechanical misalignments. Additionally, a computationally preferable calibration algorithm based on the two-face measurements is introduced. In the end, the calibration results are discussed, highlighting all necessary prerequisites for the scanner calibration from a single scanner station.

## 1. Introduction

Nowadays, commercially available panoramic terrestrial laser scanners (TLS) can reach a point accuracy in the order of millimeters over their full measuring range under optimal conditions. That makes them an interesting option for highly demanding engineering tasks, such as structural deformation monitoring. For example, deformation monitoring of dams, tunnels and radio telescopes can be found in literature [1,2,3]. In order to achieve the required measurement quality, manufacturers put considerable effort on the production and assembly of all instrument components. However, these processes are not perfect and remaining mechanical misalignments need to be modeled mathematically. That is achieved by a comprehensive factory calibration (e.g., [4]). In general, manufacturers do not provide complete information about the functional relations between the remaining mechanical misalignments and the observations, the number of relevant misalignments, as well as the magnitude and precision of the parameters describing those misalignments. This data is treated as a company secret.

That ambiguity poses a significant problem when it comes to the user recalibration of the instrument. At the time of purchase, laser scanners are expected to be free of systematic errors caused by mechanical misalignments. Additionally, their measurement quality should be consistent with the description given in the manufacturers specifications. However, many factors can influence the performance of the particular scanner, such as long-term utilization, suffered stresses and extreme atmospheric conditions. Due to that, instruments must be tested and recalibrated at certain time intervals in order to maintain the declared measurement quality. So far, the only reliable way to achieve that is by sending the instrument back to the manufacturers to repeat the factory calibration, which is a time consuming and financially burdening event.

There are certain alternatives, but they lack in comprehensiveness and reliability. For example, some manufacturers like Leica Geosystems and FARO Inc. provide user calibration approaches, which can reduce systematic errors in the measurements due to misalignments to some extent (e.g., Leica’s “Check and Adjust” and Faro’s “On-site compensation”). However, those approaches do not provide detailed information about all estimated parameters, their precision and influence on the resulting point cloud.

In the last decade, several publications showed considerable effort to overcome these problems. Their main aim was to provide a standardized, reproducible approach for the user calibration of laser scanners. However, that standardized approach is still missing and this paper continues the work on the presented topic. A short overview can be found in [5], while all relevant publications are specified in the subsequent section (Section 1.1). Furtherly, Section 1.2 describes the aim of the study.

### 1.1. Previous Work

So far, several different user-oriented calibration approaches have been proven quite successful. Most of them are considered self-calibration approaches, regarding the fact that all the calibration parameters describing measurement errors are determined simultaneously and without the need of a special facility with a dedicated test field. If some reference values for the test field are introduced, than it is no longer self-calibration, but rather system calibration [6]. In this work, the term system calibration is preferred for more general discussions, because it includes both self-calibration and calibration with eventual reference values. However, it is important to note that the experiment within the paper was conducted as a self-calibration.

Primarily, these calibration approaches can be separated according to the objects used for the calibration. Most commonly used objects are specialized scanner targets in the form of intensity-based planar targets or spheres [7,8,9,10,11]. Additionally, many researches use planar objects either dedicated to the task or found on the calibration scene [12,13,14,15,16]. Other objects used for laser scanner calibration are cylinders [17] and a paraboloid [18]. All of the mentioned approaches successfully reduced most of the systematic errors in the measurements.

In these studies, different authors used different calibration parameters to describe systematic errors in scanner measurements. Most of the parameter models are based on the total station model due to the similar construction of those two instruments. Some researchers used a simplified total station model [8,10,19,20], some more comprehensive models [18,21], and some used a total station model extended with empirically observed and defined calibration parameters [7,11,22,23,24]. As an alternative, Ref. [25] used an extended sensor model of panoramic cameras.

Attempts of analyzing effects of the genuine TLS misalignments on the measurement data can be found in [5,26,27]. It was confirmed by [28] that a part of the misalignments in the TLS can indeed be described by a total station model. However, using solely the total station model is overly simplified for such a complex instrument like TLS. The first comprehensive description of the functional, or geometrical, relations between the mechanical misalignments and the observations in the TLS was described in [29]. This calibration parameter model was proven valid for the majority of the panoramic laser scanners in [30], where several major manufacturing companies tested their approach and declared it functional. In contrast to the all previously mentioned works, this model was used for the component calibration of the laser scanners, meaning that all relevant calibration parameters are determined in a stepwise manner. However, their implementation in the TLS system calibration was not described in detail yet.

### 1.2. Aim of this Study

There are three main aims of this study that are expected to contribute to the further enhancement of the user oriented TLS calibration approaches:

- The first aim is to prove that in the case of the panoramic laser scanners most of the calibration parameters can be estimated from a single scanner station, without a need for any reference information. Therefore, substantial time savings can be achieved, and all targets can be optimally oriented for a single scanner station. This eliminates the need to use measurements compromised by steep incidence angles leading to larger errors [31,32]. Moreover, it will be discussed that even all relevant calibration parameters can be estimated, if an additional reference information is introduced.
- The second aim is to describe an adaptation and implementation of the mechanically interpretable calibration parameters from [29] to the calibration algorithms described in the Section 2.3 and Section 2.5. This implementation is not straightforward and it is discussed in detail in the subsection titled Implementation of Section 2.2. The motivation for implementing these parameters is the presumption that using the mechanically explainable parameters will lead to their better stability and reusability. Namely, the stability of the parameters usually used in the user oriented self-calibration (also system calibration) approaches is still not adequately investigated, and could be questioned, as for example in [33]. This presumption was not tested within the conducted experiment, but it is the part of the ongoing investigation and will be incorporated in future publications.
- Third, the new angular parameterization is introduced for the implicit implementation of two-face measurements. This led to a development of the new calibration algorithm based on the scanning of all targets in two faces of the instrument. It can be used to estimate most of the relevant calibration parameters with a simple measurement setup and from a single scanner station. This makes it an interesting solution for a quick instrument check-up prior to the higher demanding field tasks.

Our premises are tested through empirical experiments, which took place in a large machine hall. The large facility was utilized in order to increase the sensitivity of the calibration approach. Namely, most of the parameters used are angular parameters, and their effect is increases linearly with distance. Reference estimates of the calibration parameters are obtained through an established self-calibration approach based on a network of several scanner stations (see e.g., [7]). The approach is briefly described in Section 2.3. Additionally, the established geometry of the network of targets was validated through a simulation experiment in order to prove its possibility to estimate all relevant calibration parameters. Finally, we discuss all of the results and necessary prerequisites for a successful calibration from a single scanner station. The paper is organized as it follows: Section 2 describes the theoretical background of this work. Section 3 reviews the data acquisition and processing. In Section 4, all the results are presented together with the corresponding discussion. Finally, the conclusions of this research are drawn in Section 5.

## 2. Theoretical Background

This section introduces all relevant terms and mathematical relations needed for the complete understanding of the conducted experiment. Section 2.1 explains the geometry of a typical panoramic terrestrial laser scanner and the angular parameterization used. Section 2.2 introduces the calibration parameters adopted in this research. Section 2.3 and Section 2.4 shortly describe the used functional and stochastic models of the target-based self-calibration. Section 2.5 describes the two-face adjustment, an alternative approach to calibrate panoramic laser scanners. Finally, Section 2.6 explains the statistical test used in this research.

### 2.1. Instrument Geometry and Angular Parameterization

**Figure 1:**
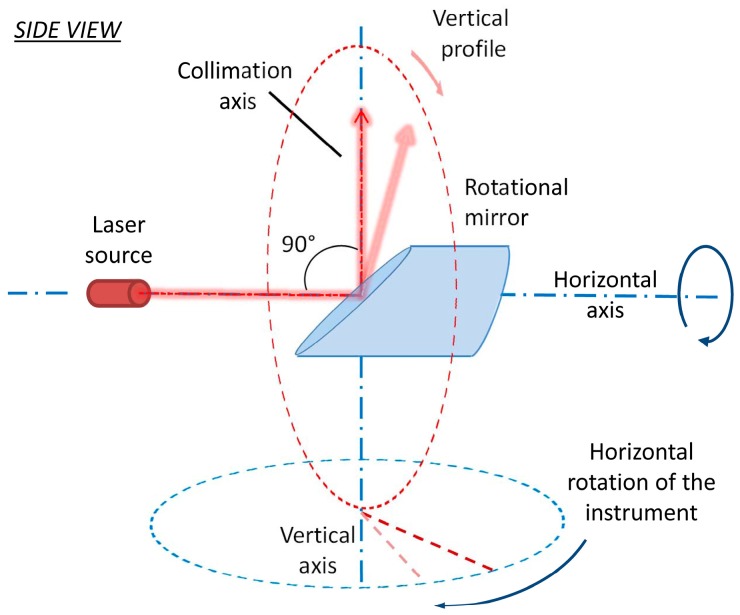
Perfect panoramic terrestrial laser scanner geometry.

Figure 1 represents the perfect geometry of the panoramic terrestrial laser scanner. The laser source fires the laser beam on the center of the rotational mirror. The rotational mirror is placed on its mount and it is inclined in the way that it intersects the secondary rotational axis with an angle of 45°. The laser beam is reflected in the direction of a measured point forming an angle of 90°. The mirror rotates around the primary rotational axis realizing the profile measurements. Additionally, the whole instrument revolves around the secondary rotational axis. The central point of the measuring system is the middle point of the mirror. The primary rotational axis is defined as a shaft of the rotating mirror. In the perfect case, it is a line intersecting both middle point of the mirror and the laser source. The secondary rotational axis is defined by the main rotational shaft of the instrument and in the perfect case it intersects the middle point of the mirror. Finally, the third or collimation axis is perpendicular to the primary axis. It is defined by the line passing through the middle point of the mirror and spanning in the direction of the reflected part of the laser beam. At the beginning of each profile measurement, the collimation axis is coincident with the secondary axis. Panoramic laser scanners are usually used in the upright position and leveled within the horizontal plane by using an inbuilt compensator. Consequently, the primary axis is usually horizontal in space, and the secondary axis is vertical. Thus, for the sake of simplicity, these axes will be henceforth referred to as the horizontal and the vertical axis.

The panoramic scanner assembly allows making two different scans of the whole surrounding from the same nominal position. Only the small conical volume directly underneath the instrument is not covered. The availability of both scans is only a question of the in-built software. In the first scan, the whole instrument rotates for half of a circle around the vertical axis. In the same time, the mirror makes full circles while measuring successive vertical profiles. We describe the motion of the mirror with the vertical angles from 0° to 360°, with 0° being realized when the scanner measures points in zenith. Further, we describe the motion of the instrument with the horizontal angles from 0° to 360°, with 0° arbitrarily defined on each scanner station. Therefore, for the first scan, the registered horizontal angles range between 0° and 180°, while the vertical angles range between 0° and 360° (with the section from 135° to 225° under the scanner). In the second scan, the instrument completes the circle around the vertical axis and this time, it registers the horizontal angles with values from 180° to 360°, while the vertical angles are again from 0° to 360°. For these two consecutive scans, we will furtherly use terms: two-face measurements or measurements in two faces.

This way, in each scan, the points are measured both in the front and the back of the instrument. The points measured in the front of the instrument are usually called first face, front face, or first layer points, while the opposite points are called second face, back face or second layer points [20]. We will use the terms first and second face of the instrument and we will assign values from 0° to 180° for the vertical angles of points in the first face, and values from 180° to 360° for the vertical angles of points in the second face. Therefore, the term face depends on the vertical angle value or the mirror position at the moment of measuring. One full 3D scan contains some points in the first and some in the second face. In the consecutive scan, the surrounding is scanned for the second time and this time the points scanned in the first and second face will be opposite. For the first scan and second scan, we will use the term cycles and we will assign values from 0° to 180° for horizontal angles of the first cycle and from 180° to 360° for horizontal angles of the second cycle. Therefore, the term cycle depends on the horizontal angle value or on the instrument rotation with respect to the vertical axis. The result of these consecutive scans is that every object in the field is measured in both faces and both cycles, providing valuable information for estimating the calibration parameters.

The final outputs of both scans are Cartesian coordinates of all measured points in the local reference system of the scanner. They are derived from the measured range, horizontal and vertical angle. The scanner initializes its local 3D Cartesian coordinate system, once it is set on one scanner station. Both scans (first and second cycle) share the same coordinate system. Thus, the corresponding points from the two consecutive scans will share the same Cartesian coordinates, even though they are derived from the different set of polar coordinates.

**Figure 2:**
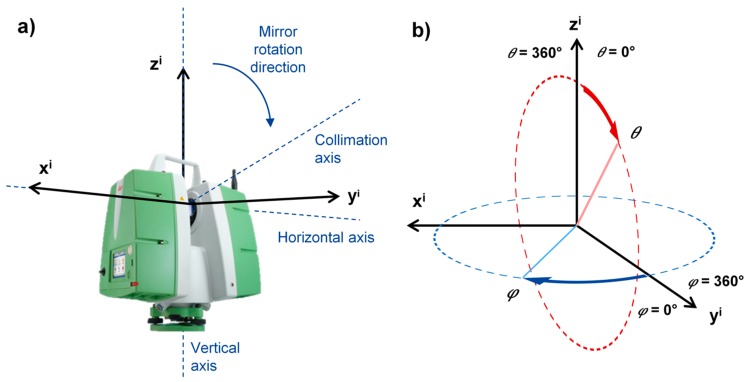
(**a**) Local Cartesian coordinate system of the scanner with a respect to the main instrument axes; (**b**) Local coordinate system of the scanner transformed to the polar coordinates.

Every instrument defines its local coordinate system a little bit differently. In this case, the Leica Scan Station P20 defines a right handed coordinate system (Figure 2a). The *Z* axis is coincident with the main vertical rotational shaft with the positive direction towards the top of the instrument. The *Y* and *X* axes describe a plane perpendicular to the *Z* axis containing the primary rotational axis of the instrument. The *Y* axis lies in the plane described by the first vertical profile scanned during the first scan (first cycle), while the *X* axis complements the right handed system.

The conversion of the described Cartesian coordinates to the polar coordinate system is needed in order to estimate meaningful calibration parameters. The polar coordinate system defined in this project is depicted in Figure 2b. The horizontal angles rotate clockwise, with the 0° on the *Y* axis. That definition describes the motion of the used instrument in the physical reality. The vertical angles describe the direction of the mirror rotation while measuring vertical profiles.

In order to extract the polar coordinates describing the actual position of the instrument in the moment of measuring, the following relations are used:

(1) $$r_{j}^{i}=\sqrt{{x_{j}^{i}}^{2}+{y_{j}^{i}}^{2}+{z_{j}^{i}}^{2}}$$

(2) $$\varphi_{j}^{i}=\arctan\left(\frac{x_{j}^{i}}{y_{j}^{i}}\right)$$

(3) $$\theta_{j}^{i}=\arccos\left(\frac{z_{j}^{i}}{r_{j}^{i}}\right)$$

where *i* = 1, 2, …, *s*; *j* = 1, 2, …, *p*; *s* and *p* are total numbers of scanner stations and targets used in the experiment, $r_{j}^{i},\varphi_{j}^{i},\theta_{j}^{i}$ are the measured ranges, horizontal and vertical angles and $x_{j}^{i}$, $y_{j}^{i}$, $z_{j}^{i}$ are the Cartesian coordinates of the measured points stored in the scanner. In the first cycle, the value of 180° is added to the horizontal angles if the calculated angle is negative. This is the case because the scanner actually performed only half of the rotation around the vertical axis, using the same horizontal angle readings for points measured both in the first and the second face. In the second cycle, the scanner uses a new set of diagonally opposite angular readings for the horizontal angles. Therefore, in the second cycle, the value of 180° is added if the calculated angle is positive and 360° if the angle is negative. The position of the mirror is easily tracked with the $x_{j}^{i}$ coordinate value. If this coordinate in the first cycle is negative, the calculated vertical angle is subtracted from 360°. In the second cycle, the case is opposite. If the $x_{j}^{i}$ coordinate is positive, the calculated vertical angle is subtracted from 360°.

This angular parametrization allows an easy implicit inclusion of measurements of the same object in two different faces (and cycles) from the same nominal position of the instrument. Additionally, it eases incorporating a new set of calibration parameters, what is described in the following section.

### 2.2. Calibration Parameters

Panoramic terrestrial laser scanners are similar to a total station to a certain extent. Like in the total station, a TLS has three main axes and not achieving their orthogonality produces measurement errors. However, because of the complex structure of the instrument, the number of possible misalignments in a TLS is larger than in a total station [5,21]. As already mentioned in Section 1.2, this research uses the mechanical parameters adopted from [29] and readers are encouraged to consult that reference for a better understanding of the error sources. However, for completeness, a short description of the mechanical parameters is provided (Table 1). The description is followed by a detailed explanation of the incorporation of these calibration parameters into the system calibration approach.

#### 2.2.1. Laser Source

**Figure 3:**
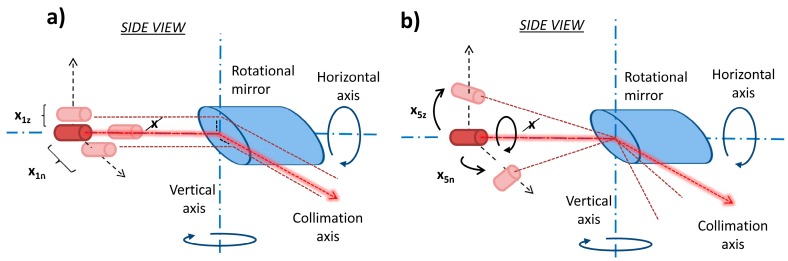
Laser source related mechanical misalignments: (**a**) laser source offset; (**b**) laser source tilt.

In contrast to the perfect case, the laser source is misplaced to some extent in every instrument. It can be translated and/or rotated relative to the rotating mirror leading to systematic errors in the acquired measurements. The laser source can be translated in three directions: in direction of the instrument’s vertical axis, in direction of the rotating mirror and in direction perpendicular to the mirror (Figure 3a). The translation in the direction of the mirror is either removed by the reference range measurement or it is absorbed in the rangefinder offset parameter and therefore, it is not mentioned henceforth. The remaining two translations affect both the measured horizontal and vertical angles. Therefore, they are modelled by the *x*_1*n*_ and *x*_1*z*_ calibration parameters. Similar to the translation case, the laser source can rotate around three axes. While the rotation around the horizontal axis does not influence the measurements, the rotations around the vertical axis and collimation axis influence the measurements (Figure 3b). These misalignments are denoted as parameters *x*_5*n*_ and *x*_5*z*_.

#### 2.2.2. Rotating Mirror

**Figure 4:**
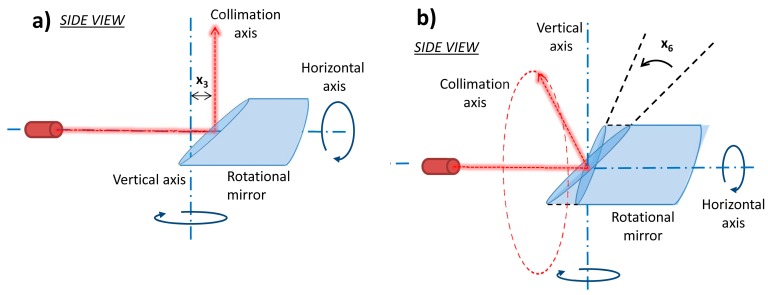
Rotational mirror related mechanical misalignments: (**a**) mirror offset; (**b**) mirror tilt.

In addition to the laser source misplacements, two cases of mirror errors can also affect the measured values. In both of these cases, the mirror rotates around the horizontal axis and the horizontal axis is positioned as in the perfect case. The first one is the offset of the mirror along the horizontal axis affecting both measured range and horizontal angle (Figure 4a) and it is denoted as *x*_3_. The influence on the range is successfully removed in most of the laser scanners by the reference range measurement or it is absorbed in the rangefinder offset parameter, so it is not included in the functional model of the range related calibration parameters (Equation (4)). The second error is the mirror tilt (Figure 4b), denoted as *x*_6_. This error describes the case when the mirror is incorrectly placed on its mount and because of that it does not intersect the vertical axis with the inclination of 45°. The term influences only the measured horizontal angles.

#### 2.2.3. Primary Rotational or Horizontal Axis

**Figure 5:**
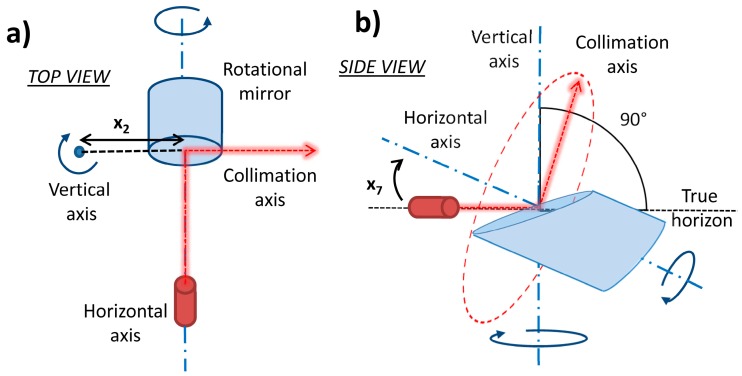
Horizontal axis related mechanical misalignments: (**a**) axis offset; (**b**) axis tilt.

Separate cases of misalignments are the offset and the tilt of the whole horizontal axis together with the mirror on its mount. In the first case, the horizontal axis still lies in the horizontal plane and it is perpendicular to the vertical axis. However, it does not intersect with the vertical axis, but passes nearby (Figure 5a). This misalignment affects both ranges and vertical angles and it is denoted by the parameter *x*_2_. The second case is equal to the horizontal axis error in the total station, horizontal and vertical axes do not intersect forming an angle of 90° (Figure 5b). This case is modelled with the *x*_7_ parameter. The difference between the latter error and the error represented in Figure 4b is in the different position of the horizontal axis in space, which conditions the different path of the laser beam. Hence, it has different effect on the measured angles.

#### 2.2.4. Total Station Related Parameters

All the other mechanical misalignments are equivalent to the misalignments in the total station and will not be described in detail. Those are namely the vertical index offset (*x*_4_), rangefinder offset (*x*_10_), and encoder related errors. A comparison between the total station model of parameters [21] and mechanical model is given in Table 1. The main focus of the research is placed on the imperfection of the instrument components assembly. Therefore, an extensive rangefinder calibration was not included. As a result, calibration parameters such as rangefinder scale and cyclic errors are omitted.

#### 2.2.5. Implementation

The set of parameters presented in the left part of Table 1 needs an optimization for the system calibration approach. Some of the parameters have completely the same effect on the measurements. Hence, they cannot be estimated separately and they need to be combined. In this paper, two cases of the parameter combinations are required. The parameters for the vertical beam tilt (*x*_5*z*_) and the horizontal axis tilt (*x*_7_) are combined in one (*x*_5*z*−7_) due to the same functional definition. Avoiding this operation would lead to a poor condition number and to a singularity in the normal equations. We would like to specially highlight the case of the parameters *x*_1*n*_ and *x*_2_. Although, from the theoretical stand point, they could be separately estimated without singularity in the normal equations, we strongly recommend introducing the combined parameter *x*_1*n*+2_. Leaving out this parameter leads to a significant bias in the final estimates of the calibration parameters. This is explained in detail in Section 4.1 through the conducted simulations.

Additionally, in theory, the horizontal beam tilt (*x*_5*n*_) affects both horizontal and vertical angles, but in practice the case is different. As it was proven in [28], this error term is partially absorbed in the exterior orientation parameters, more precisely in the rotation angle around the vertical axis. Consequently, there is no influence on the horizontal angles and this parameter can be omitted from the horizontal angles equation. Furthermore, the used TLS exploits four orthogonal reading heads for the calculation of both horizontal and vertical angles [4]. Therefore, it can be presumed that all encoder related errors (Table 1) are averaged and removed. As a result, they are omitted from the functional model.

The final functional relations for the range (r), horizontal (φ) and vertical (θ) angular measurement corrections are described as follows:

(4) $$\Delta r_{j}^{i}=x_{2}\sin\left(\theta_{j}^{i}\right)+x_{10}+v_{r_{j}^{i}}$$

(5) $$\Delta \varphi_{j}^{i}=\frac{x_{1z}}{r_{j}^{i}\tan\left(\theta_{j}^{i}\right)}+\frac{x_{3}}{r_{j}^{i}\sin\left(\theta_{j}^{i}\right)}+\frac{x_{5z-7}}{\tan\left(\theta_{j}^{i}\right)}+\frac{2x_{6}}{\sin\left(\theta_{j}^{i}\right)}+\frac{x_{1n}}{r_{j}^{i}}+v_{\varphi_{j}^{i}}$$

(6) $$\Delta \theta_{j}^{i}=\frac{x_{1n+2}\cos\left(\theta_{j}^{i}\right)}{r_{j}^{i}}+x_{4}+x_{5n}\cos\left(\theta_{j}^{i}\right)-\frac{x_{1z}\sin\left(\theta_{j}^{i}\right)}{r_{j}^{i}}-x_{5z}\sin\left(\theta_{j}^{i}\right)+v_{\theta_{j}^{i}}$$

where $\Delta r_{j}^{i},\Delta \varphi_{j}^{i},\Delta \theta_{j}^{i}$ are disagreements in the calibration adjustment, $r_{j}^{i},\varphi_{j}^{i},\theta_{j}^{i}$ are the measurements in polar coordinates, $x_{1n/2/}$*_…_* terms are the calibration parameters describing the systematic errors and $v_{r_{j}^{i}}$, $v_{\varphi_{j}^{i}},\;v_{\theta_{j}^{i}}$ are the adjustment residuals describing the random errors. As it can be seen from the equations, this paper uses a set of 11 calibration parameters in order to describe all relevant mechanical misalignments. It is important to note that the multiplication factor *k* used in the referent literature [29] was omitted herein due to the different parametrization of the polar measurements.

#### 2.2.6. Two-Face Sensitivity

Some of these parameters can be estimated by observing only one target from the same position with two-face measurements, if the measurement noise is disregarded. These parameters are referred as the parameters sensitive to two-face measurements or the two-face sensitive parameters. This attribute directly arises from their functional definition, hence from the scanner geometry. In order to be two-face sensitive, the influence of the parameter should change its sign in two-face measurements for the ranges and horizontal angles (Equations (4) and (5)). On the contrary, it should retain a constant sign in both faces in the case of vertical angles (Equation (6)). This namely depends on the way of calculating the difference of corresponding measurements in two faces. The difference for ranges, horizontal and vertical angles are calculated as follows:

(7) $$dr=(r_{2}+\Delta r)-\left(r_{1}+\Delta r\right)$$

(8) $$d\varphi =[(\varphi_{2}+\Delta \varphi )-180{}^{\circ}]-(\varphi_{1}+\Delta \varphi )$$

(9) $$d\theta =\left[360{}^{\circ}-(\theta_{2}+\Delta \theta \right)]-\left(\theta_{1}+\Delta \theta \right)=360{}^{\circ}-\left[(\theta_{2}+\Delta \theta \right)+\left(\theta_{1}+\Delta \theta \right)]$$

where $r_{2},\varphi_{2},\theta_{2}$ are measurements in the second face (or cycle) and $r_{1},\varphi_{1},\theta_{1}$ are measurements in the first face (or cycle). This way of calculating the difference of corresponding two-face measurements is usually used for the total station observations and can be found in [34]. The two-face sensitivity is easily observable in Equation (9). If the parameter influence changes the sign from +Δθ in one face to −Δθ in other face, it is removed from the difference of the corresponding vertical angles. Hence, the parameter is not determinable using this information.

A total of nine out of 11 parameters from Equations (4)–(6) can be estimated using only two-face measurements. Most of the parameters are two-face sensitive in all equations. However, the parameter *x*_1*z*_ is sensitive in the horizontal angle equation, while it is not in the vertical angle equation. For the parameter *x*_1*n*_, the case is opposite. It can be estimated in the vertical angle equation within the parameter *x*_1*n*+2_ and separated later (detailed explanation in Section 4.1). Therefore, we can say that the parameters *x*_1*z*_ and *x*_1*n*_ are partially two-face sensitive.

The value, or the influence, of the parameter *x*_5*z*_ is contained within the parameter *x*_5*z*−7_, which is a two face sensitive parameter. However, due to the same functional definition and the lack of additional information, like in the case of parameters *x***_1*n*_** and *x*_2_, the value of the parameter *x*_5*z*_ cannot be separated from the parameter *x*_5*z*−7_. Hence, the value of the parameter *x*_5*z*_ cannot be estimated from two-face measurements and henceforth, we treat this parameter as the parameter insensitive to two face measurements.

**Figure 6:**
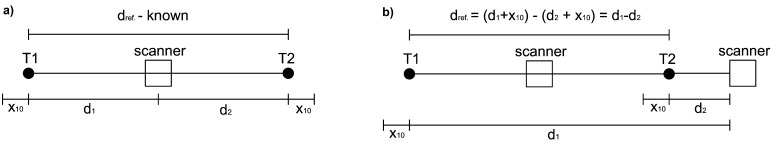
Estimating rangefinder offset parameter *x*_10_: (**a**) with known reference; (**b**) without known reference.

The only two parameters that cannot be estimated from two-face measurements are the range-finder offset (*x*_10_) and vertical beam tilt (*x*_5*z*_). The parameters should be at least partially two-face sensitive in order to be estimated from a single scanner station without any reference. Otherwise, an additional reference value is mandatory for the complete TLS calibration. This will be explained on a simple example of the rangefinder offset parameter. The rangefinder offset retains the same sign in each of the two-face measurements. Therefore, it is not contained in the difference dr and it cannot be estimated by observing one target from one location without a reference. There are two straightforward ways of estimating the parameter *x*_10_ depicted in Figure 6 [35].

For both cases, the scanner should be placed in the middle between two targets and in line with them. The first one (Figure 6a) is when the distance between the targets is measured with a reference instrument of the higher nominal accuracy. Therefore, we presume that the true value of the measured distance is known a priori and the parameter *x*_10_ equals half of the difference between the true value and the value measured with the scanner. In the second case, we do not know the true value of the distance between targets. For this instance, an additional scan is required, with the scanner placed outside of the measured distance while still being placed in line with the targets (Figure 6b). The distance estimated from the second scan will not be influenced by the parameter *x*_10_ and, therefore, it can be used as a reference distance, in the same manner as in the previous example.

Usual self-calibration approaches (e.g., [7]), requiring scans from several locations, draw the information from the latter case. However, the scanner is never in line with the targets when placed outside the measured distance, due to the limits of the network design. This is mostly compensated with a high measurement redundancy. In the case of calibrating the scanner from one scanner station, the described approach cannot be employed. Hence, the reference distance is a mandatory prerequisite for the successful estimation of the parameter *x*_10_. A similar logic can be applied for the parameter *x*_5*z*_. Therefore, the reference distance is required for its successful estimation. The optimal placement of the reference distance for estimating the parameter *x*_5*z*_ is a part of the ongoing investigation.

### 2.3. Functional Model of the System Calibration (Self-Calibration)

Our study mainly relies on the most common self-calibration approach using dedicated intensity (black and white) planar targets. This well-established calibration approach is used to obtain the reference calibration results, which are later compared with the proposed calibration from a single scanner station. Additionally, the new algorithm described in Section 2.5 is obtained by the simplification of the algorithm described in this section.

The scanner self-calibration is derived from the bundle adjustment, which is often used for calibrating digital cameras. It was introduced for the first time in the TLS calibration in [36]. The root of the approach is a rigid body transformation. In this instance, the transformation is used for the simultaneous registration of several local coordinate systems to a single reference system. It relies on the redundant distinct point features derived from the 3D point cloud of the observed scene, which are mutual to each local coordinate system. Points are derived from the point cloud using specialized intensity based TLS targets. Local coordinate systems are realized by the instrument on each scanner station used in the experiment (Section 2.1). The realization of the reference coordinate system depends namely on the selected datum definition, while its approximation is usually arbitrarily chosen.

The only addition to this simultaneous registration is a presumption that the disagreements in the registration, the measurement residuals, are not solely random, but rather a combination of random and systematic influences. A part of these systematic influences originates from the mechanical misalignments of the instrument. The main aim of the adjustment is to estimate the calibration parameters that model those misalignments (Equations (4)–(6)).

Due to the redundancy of the point features, the described approach is mathematically realized as a least squares adjustment. In this paper, we adopted the adjustment based on Gauss-Helmert model [37], which was introduced for the TLS calibration in [38], in an extended from. The functional model is defined as follows:

(10) $$f_{j}^{i}=R_{\left(k,\phi ,\omega \right)}^{i}xyz_{j}^{i}+T_{\left(X,Y,Z\right)}^{i}-XYZ_{j}^{ref.}=0$$

where *i* = 1, 2, …, *s*; *j* = 1, 2, …, *p*; *s* and *p* are total numbers of scanner stations and targets used in the experiment, $R_{\left(k,\phi ,\omega \right)}^{i}$ is the rotation matrix with three rotation angles (k,ϕ,ω) around the main coordinate axes and $T_{\left(X,Y,Z\right)}^{i}$ is the translation vector in the direction of those axes. They describe position and orientation of the local coordinate system of each scanner station relative to the reference coordinate system. The vector of the measurements from the scanner station *i* to the target *j* in the local coordinate system equals:

(11) $$xyz_{j}^{i}=\left[\begin{array}{c} x_{j}^{i}\\ y_{j}^{i}\\ z_{j}^{i} \end{array}\right]=\left[\begin{array}{c} \left(r_{j}^{i}+\Delta r_{j}^{i}\right)\sin\left(\theta_{j}^{i}+\Delta \theta_{j}^{i}\right)\sin\left(\varphi_{j}^{i}+\Delta \varphi_{j}^{i}\right)\\ \left(r_{j}^{i}+\Delta r_{j}^{i}\right)\sin\left(\theta_{j}^{i}+\Delta \theta_{j}^{i}\right)\cos\left(\varphi_{j}^{i}+\Delta \varphi_{j}^{i}\right)\\ \left(r_{j}^{i}+\Delta r_{j}^{i}\right)\cos\left(\theta_{j}^{i}+\Delta \theta_{j}^{i}\right) \end{array}\right],$$

where values $\Delta r_{j}^{i},\Delta \varphi_{j}^{i},\Delta \theta_{j}^{i}$ contain the correction for both random errors, described by the adjustment residuals, and mentioned systematic errors, described by the estimated calibration parameters (Equations (4)–(6)). $XYZ_{j}^{ref.}$ is the vector containing the final estimates of the target Cartesian coordinates in the reference coordinate system. These coordinates are homologue to the ones described with polar measurements in $xyz_{j}^{i}$.

To sum up, the main input values are the TLS measurements of the target points recalculated in polar coordinates ($xyz_{j}^{i}$), and the main output values are the final estimates of the unknown parameters. The parameters are separated in three groups. In the first group are parameters describing the rotation and translation of each scanner station, often noted in literature [7] as the exterior orientation parameters (EOPs). The second group is formed of the target Cartesian coordinates in the reference frame, usually named as object points (OPs). The last group consists of the calibration parameters (CPs) defined in Equations (4)–(6), also named as additional parameters.

The solution is computed by minimizing the sum of weighted squared residuals. Therefore, the functional model described in Equation (10) has to be linearized by a first order Taylor approximation, leading to:

(12) Bv+AΔx+w=0,

where v is the vector of residuals, Δx is the vector of reduced parameters, w is the misclosure vector, while B and A are Jacobian matrices with respect to the observations and the unknown parameters, which are computed by:

(13) $$B=\frac{\partial f_{j}^{i}}{\partial L}|x^{0},L^{0}$$

(14) $$A=\frac{\partial f_{j}^{i}}{\partial x}|x^{0},L^{0}.$$

Here, L is the vector containing scanner observations in polar coordinates, $x^{0}$ is the vector of approximated values of unknown parameters and $L^{0}$ is the vector of approximated values of the observations. In this study, we use the rigorous solution of the Gauss Helmert model. That means that besides the iterative update of the parameters’ approximate values, the estimates of the adjusted measurements are updated in each iteration as well [39].

The parameter estimates are calculated in an iterative procedure and they are given as the vector of final estimate of all unknown parameters with corresponding covariance matrix ($\Sigma_{\hat{x}\hat{x}}$):

(15) $$\hat{x}=\left[\begin{array}{c} {\hat{x}}_{EOP}\\ {\hat{x}}_{CP}\\ {\hat{x}}_{OP} \end{array}\right]=\left[\begin{array}{c} x_{EOP}^{0}\\ x_{CP}^{0}\\ x_{OP}^{0} \end{array}\right]+\left[\begin{array}{c} \Delta {\hat{x}}_{EOP}\\ \Delta {\hat{x}}_{CP}\\ \Delta {\hat{x}}_{OP} \end{array}\right]$$

where the vector $\hat{x}$ is the final estimate of the parameters, while $x_{EOP/CP/OP}^{0}$ are approximate values of the parameters. A similar adjustment algorithm can be found in [1]. The algorithm can be realized differently, as a weighted total least squares problem, which can reduce the computational time according to [40]. However, the final solution is numerically equivalent to the solution of the algorithm used in this experiment. Therefore, a weighted total least squares estimation is not further considered in this work.

### 2.4. Stochastic Model

In order to estimate unbiased and precise calibration parameters, an adequate stochastic model is required. It is common to use the data from the manufacturer’s specifications for the purpose of building the covariance matrix of scanner observations. However, when engaging the process of TLS calibration, we expect that the instrument needs a recalibration. In other words, we expect that the instrument performance is degraded in comparison to the performance in the moment of manufacturing. Additionally, specifications are usually pessimistic, they include eventual remaining systematic errors, and they do not reflect the true performance of an individual scanner. Therefore, we use the manufacturer specifications for the initial weighting only, as an initial guess, and then refine the stochastic model through the variance component estimation (VCE) for each group of observations. The VCE is integrated in the calibration algorithm which contains estimated calibration parameters. As a result, the assessed measurement precision incorporates only random errors, while the systematic errors are functionally modeled. The stochastic model is accepted when the global test is accepted [6].

The covariance matrix of the observations Σ is formed as a diagonal matrix. The correlations between the measurements are disregarded due to the lack of an appropriate correlation model [27]. The covariance matrix is introduced in the adjustment while solving the normal equations [37].

The network datum definition directly influences the values of the final covariance matrix of the estimated parameters and, therefore, influences their precision and correlations. In this work, we use an inner constrained datum, which is the most often one in the TLS calibration (see e.g., [7]). The inner constrained datum is mathematically realized by minimizing the trace of one block of the covariance matrix of the estimated parameters ($\Sigma_{\hat{x}\hat{x}}$). In the case of the TLS system calibration, that block corresponds to the estimated target coordinates. The standard form of the inner constraint matrix can be found, for example, in [6].

### 2.5. Two-Face Adjustment Algorithm

In this paper, we propose a simplified algorithm for the calibration of panoramic laser scanners, which can be achieved from a single scanner station. Usual self-calibration approaches (Section 2.3) rely on measuring an established point field from several locations with several instrument orientations in order to estimate the calibration parameters. On the contrary, the proposed two-face adjustment method is based on analyzing the difference between the measurements of all targets in the first and second face of the instrument, from the same nominal position. Even though the purpose of the algorithm is the calibration from the single station, the algorithm is able to use the measurements from multiple scanner stations. However, this possibility was not employed within this work. Hence, only the data from one scanner station was used. One attempt of estimating only three calibration parameters (based on the total station model) by using a similar approach can be found in [19].

The difference between the corresponding first and second face measurement in the ideal scanner should equal 0. Hence, that is the true or reference value. The deviation from this true value is introduced by uncalibrated mechanical misalignments and random measurement noise. This information can be used for estimating calibration parameters, if the influence of certain misalignment is larger than the noise or the noise can be averaged and removed. Therefore, the only information needed for the estimation of most calibration parameters (Section 2.2) are two-face measurements, or in other words, the corresponding measurements with different mirror and instrument positions.

The concept is adopted from the total station component calibration [34] and expanded for the case of the laser scanner. In contrary to the total station case, due to the larger number of calibration parameters, a stepwise parameter estimation or separate component calibration is not possible. Hence, the adjustment procedure based on the redundant measurements is required to estimate the most probable combination of all parameters. The adjustment is realized as the least squares minimization problem based on the Gauss-Helmert model, similar to the one described in Section 2.3. The general functional model is structured as follows:

(16) $$f^{i}=xyz_{2}^{i}-xyz_{1}^{i}=\left[\begin{array}{c} x_{2}^{i}\\ y_{2}^{i}\\ z_{2}^{i} \end{array}\right]-\left[\begin{array}{c} x_{1}^{i}\\ y_{1}^{i}\\ z_{1}^{i} \end{array}\right]=0$$

where $f^{i}$ is the triplet of functions for each target in the calibration field, while $xyz_{2}^{i}$ and $xyz_{1}^{i}$ are the vectors of scanner observations in the second and first face, equivalent to the ones in Equation (11). Therefore, the observations can be expressed as functions of the calibration parameters and polar TLS measurements obtained on the field. As explained in Section 2.2, some calibration parameters are not two-face sensitive. Hence, for the two-face adjustment algorithm, the calibration parameter equations are in the reduced form:

(17) $$\Delta r_{j}^{i}=x_{2}\sin\left(\theta_{j}^{i}\right)+v_{r_{j}^{i}}$$

(18) $$\Delta \varphi_{j}^{i}=\frac{x_{1z}}{r_{j}^{i}\tan\left(\theta_{j}^{i}\right)}+\frac{x_{3}}{r_{j}^{i}\sin\left(\theta_{j}^{i}\right)}+\frac{x_{5z-7}}{\tan\left(\theta_{j}^{i}\right)}+\frac{2x_{6}}{\sin\left(\theta_{j}^{i}\right)}+v_{\varphi_{j}^{i}}$$

(19) $$\Delta \theta_{j}^{i}=\frac{x_{1n+2}\cos\left(\theta_{j}^{i}\right)}{r_{j}^{i}}+x_{4}+x_{5n}\cos\left(\theta_{j}^{i}\right)+v_{\theta_{j}^{i}}$$

Further on, the functional model is linearized by the first order Taylor approximation, resulting in Equation (12). However, in this instance, the configuration matrix A and the vector of reduced parameters Δx are significantly simplified due to the absence of the parameters describing the scanner’s exterior orientation and the target coordinates.

The stochastic model for the scanner observations is derived empirically. It is done by analyzing the a priori standard deviation of the half of difference between two-face measurements, separately for ranges, horizontal and vertical angles:

(20) $$\sigma_{r}=\overset{s}{\underset{i=1}{\sum }}\left|\frac{r_{2}-r_{1}}{2}\right|$$

(21) $$\sigma_{\varphi }=\overset{s}{\underset{i=1}{\sum }}\left|\frac{(\varphi_{2}-180{}^{\circ})-\varphi_{1}}{2}\right|$$

(22) $$\sigma_{\theta }=\overset{s}{\underset{i=1}{\sum }}\left|\frac{360{}^{\circ}-(\theta_{2}+\theta_{1})}{2}\right|$$

This way, the measurement precision accomplished on the field is used, rather than values from the manufacturer’s specifications. The covariance matrix of observations Σ is again formed as a diagonal matrix, as explained in Section 2.4. The final solution of the adjustment is iteratively computed and again, the rigorous solution of Gauss-Helmert model is applied, as explained in Section 2.3.

Therefore, the proposed algorithm can be used for almost complete calibration of the panoramic scanners using only two scans from a single scanner station. The parameters missing in Equations (17)–(19) are the rangefinder offset *x*_10_, the vertical beam tilt *x*_5*z*_ and the horizontal beam offset *x*_1*n*_. While *x*_10_ and *x*_5*z*_ cannot be estimated using the proposed approach, *x*_1*n*_ can be derived a posteriori. It is achieved by subtracting the value of the parameter *x*_2_ estimated in Equation (17) from the value of the combined parameter *x*_1*n*+2_ estimated in Equation (19). The detailed explanation is given in Section 4.1.

The main advantage of the algorithm lies in reducing the number of unknowns from couple of hundreds, depending on the network geometry, to nine. Besides easier computation and speed, this is very useful for the analysis of partial redundancies of the measurements, without need of a mathematical reduction of irrelevant unknowns. Thus, a planning of the calibration field, in the view of the optimal target selection, and the sensitivity analysis for the outlier detection are enhanced [41]. In order to test the validity of the proposed algorithm, the results of the calibration are compared with the accepted system calibration results in Section 4.2.

### 2.6. Congruency Test

The main statistical test used within this research is the congruency test. The objective of the congruency test is to detect whether two groups of estimated parameters are significantly different. It is usually used for evaluating the consistency of the network of points observed in two epochs [42]. In this paper, it serves two purposes. The first one is to estimate if the parameters estimated with two different approaches are significantly different. If the test is rejected, the two different approaches cannot provide the same results. The test statistics $T_{C}$ is compared with the value from the Fisher distribution:

(23) $$T_{C}=\frac{1}{h}{\left({\hat{x}}_{2}-{\hat{x}}_{1}\right)}^{T}{\left(\Sigma_{\hat{x}\hat{x}}^{2}+\Sigma_{\hat{x}\hat{x}}^{1}\right)}^{-}\;\left({\hat{x}}_{2}-{\hat{x}}_{1}\right)\leq F_{\left(h,r,1-\alpha \right)}$$

where h is the number of estimated parameters, ${\hat{x}}_{2}$ and ${\hat{x}}_{1}$ are the vectors of parameter estimated from the two different adjustments, $\Sigma_{\hat{x}\hat{x}}^{2}$ and $\Sigma_{\hat{x}\hat{x}}^{1}$ are the covariance matrices of the estimated parameters, r in this case is the sum of redundancies of the both adjustments and α is the chosen error probability factor, in this case α = 5% [42].

Additionally, the test is used to evaluate the quality of the estimated parameters. It is used in the simulation process to validate the sensitivity of the chosen network geometry (Section 4.1). The estimated parameters are compared with their known true values. In this case, ${\hat{x}}_{1}=x_{1}$, $\Sigma_{\hat{x}\hat{x}}^{1}=0$, and r=∞ in Equation (24). If the test is rejected, the estimated parameters are significantly different from their true values. Hence, the network geometry used in the experiment is not sensitive enough to provide unbiased estimates of all relevant calibration parameters.

## 3. Experiment

The experiment is divided into the empirical experiment described in Section 3.1 and the simulation experiment described in Section 3.2. The simulation experiment is used to validate the established network geometry, while the empirical experiment is used to test the main premises of this work. One premise is that most of the calibration parameters can be successfully estimated from only one scanner station and the other one is that a simple two-face adjustment can provide similar results as the usual self-calibration algorithm. Section 3.3 gives a short overview of the data processing.

### 3.1. Empirical Experiment

Section 3.1.1 provides a description of the instrument used in the experiment, Section 3.1.2 describes the established calibration field, while Section 3.1.3 explains the scanning process. Finally, Section 3.1.4 shortly summarizes the preprocessing of the scanner measurements.

#### 3.1.1. Instrument

A Leica ScanStation P20 instrument was used for the data acquisition in the field. It is a highly accurate panoramic laser scanner with the maximum range of 120 m and field of view of 360° × 270°. The nominal accuracy of the angular measurements is 8ʺ while the accuracy of the range measurements is better than 2 mm over the whole measuring range [43]. In the experiment, a maximal resolution (0.8 mm at 10 m) and the quality level 1 were used, resulting in a scanning time of 54 min for the whole 3D volume. The maximal resolution was necessary due to the large dimensions of the calibration field. Quality level 1 was chosen in order to reduce the influence of additional computations on the measurement data. Namely, the quality level denotes how many distinct point measurements are averaged to provide coordinates of one point in the point cloud, with the level 1 being the lowest level, without any averaging.

#### 3.1.2. Calibration Field

**Figure 7:**
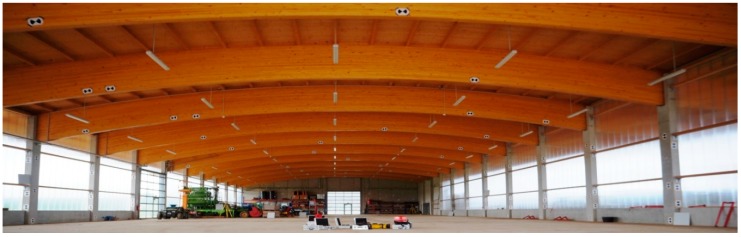
Calibration hall.

The measurements were carried out in May 2016 in a large machine hall owned by the University of Bonn. To the authors’ knowledge, it is the largest indoor facility ever used for this purpose. The dimensions of the hall are approximately 71.5 × 25.0 × 8.5 m and it was used in order to increase the sensitivity of the calibration. As it can be seen in Equations (4)–(6), most of the calibration parameters are angular values and their influence on the resulting point error is increased on higher ranges. A total of 291 targets were distributed through the whole measuring volume. The target locations were conditioned by the building properties. As it can be seen in Figure 7, the building walls are made of polycarbonate sheets supported with concrete pillars. Only the back wall is fully made out of concrete. Most of the targets were placed on the pillars in order to assure a good stability and good reflective properties. The roof of the building is supported by wooden ribs being useful for achieving favorable incidence angles on higher elevations. Beside several targets placed directly above the scanner, the rest of the elevated targets were placed on the roof ribs.

Most of the targets were paper targets based on the official Leica template (Black & White—High Definition Surveying) and printed on the thick A4-size paper (177 g/m^2^). They were attached with adhesive tape on the predetermined locations on the building. The data acquisition was carried out within 24 h after the network assembly, guaranteeing the target stability. Additionally, 16 Leica “Tilt & Turn” targets, placed on tripods and magnetic holders, were incorporated in order to improve the network geometry at otherwise occluded parts in the back of the building.

**Figure 8:**
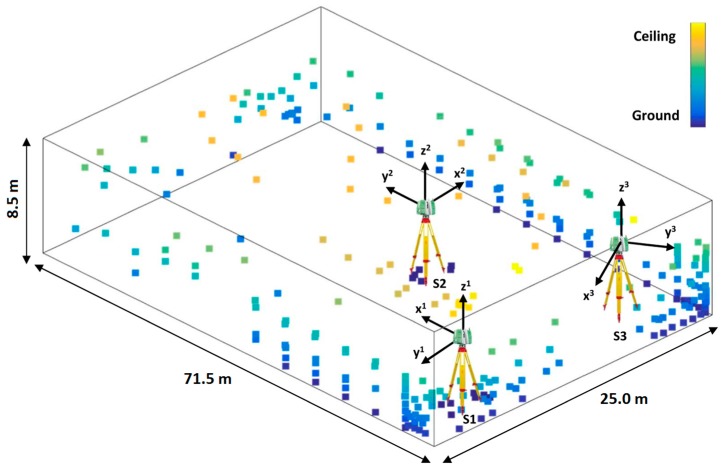
Network configuration—the scanner station locations (S1, S2, S3) with the orientation of scanner local coordinate systems and target distribution.

The achieved network configuration is visualized in Figure 8. The large horizontal dispersion of targets is evident. In contrary, the vertical distribution of targets was limited. The targets on the roof were placed selectively due to time constraint. Moreover, the targets on the floor were used only in the close proximity to the scanner in order to avoid incidence angles larger than 60°, as it was suggested in previous studies [11]. This resulted in the range of the vertical angles from 2° to 140° and from 219° to 358° (discontinuity under the tripod). As it will be presented in Section 4, the resulting vertical dispersion of targets provided a sufficient sensitivity for the good estimate of the calibration parameters. Furthermore, the obtained distance measurements range from 2.2 m to 69.1 m. It is important to note that not every target was visible from each station.

#### 3.1.3. Obtaining Scanner Measurements

The measurements were conducted from three scanner stations, indicated in Figure 8. The station locations were conditioned by the final network configuration. The main aims were achieving the best possible spatial distribution of measurements, a good overlap of measured targets between the stations and avoiding incidence angles higher than 60°. The latter condition prevented scanner stations on diametrically opposite sides, what was the common practice in previous works (e.g., [20]). As a result, an isosceles triangle spawning to the middle of the hall was selected.

All measurements have been performed within one working day. Due to time constraint, a total of five scans were made. Measurements in two cycles were conducted from the first two scanner stations, while from the third station only a scan in the first cycle was made. The orientations of the scanner were changed between the stations with the aim of reducing parameter correlations, as it was explained in [20]. The instrument was rotated for approximately 90° and 135° between the stations (Figure 8). In order to provide maximal stability of the device, a heavy duty tripod placed on the steel blocks was used on each station. The temperature and pressure were measured before each scanning and they were quite stable during the experiment (temperature around 19 °C and pressure 988 hPa).

#### 3.1.4. Preprocessing Scanner Measurements (in Leica Cyclone)

The target centers were estimated for all five scans using the software provided by manufacturer. Some targets were not successfully recognized due to bad intensity values: when targets are placed almost perfectly perpendicular to the instrument, the reflected signal is too strong to successfully estimate the center. Therefore, from the initial amount of 291, a total of 269 targets were detected. Their 3D Cartesian coordinates were recalculated in polar coordinates according to Equations (1)–(3). Five scans from three stations produced a total of 3102 measurements. This way, the measured values for the calibration algorithms are derived. Further on, all five scans were registered in the coordinate system of the first scan. This way, the reference coordinate system is defined and the approximate values for the scanner’s exterior orientation and target coordinates were estimated. This step provided all of the necessary input information required for the simulations and calibration.

### 3.2. Simulation Experiment

The main idea of the simulation experiment is to create an independent set of measurements deliberately influenced by the fictive mechanical misalignments of the known magnitude. The purpose is to test if all systematic errors (modeled by calibration parameters) can indeed be estimated unbiasedly based on the given network configuration. The congruency test is given in Section 2.6. The simulation experiment is based on the data acquired on the field. The new set of measurements is simulated from the target coordinates and the scanner exterior orientation parameters estimated for each scanner station.

Every simulated measurement has its equivalent in the empirical experiment. We propagated the influence of the simulated mechanical misalignments on the new measurements, according to the Equations (4)–(6). We chose realistic values in the same order of magnitudes as the expected measurement noise. All of the angular parameters describing the simulated misalignments were given the values of −8ʺ and all of the linear parameters were given the values of −0.2 mm. There are only two exceptions. The rangefinder offset parameter *x*_10_ was set to −2 mm, because it is expected to have the larger value. Also, the parameter *x*_7_ was set to 8ʺ in order to avoid mutual elimination of the parameters *x*_7_ and *x*_5*z*_, see Equation (5).

After the effect of the misalignments was applied, the synthetic noise according to the manufacturer’s specifications was also introduced. Angular noise was set to be 8ʺ, while the linear noise was derived by the simple analysis of the expected range noise. From the simple linear regression, based on the expected noise on certain distance from the scanner (manufacturer’s specifications), we derived the noise values of 0.2 mm + 12 ppm. This way we created an independent set of measurements influenced by the synthetic mechanical misalignments of the known value.

### 3.3. Data Processing

First, we estimated the simulated calibration parameters from the full network configuration (Figure 8) using the self-calibration algorithm described in Section 2.3 and Section 2.4. The estimated values are statistically tested (Equation (24)) in order to validate the network configuration.

Then, the same adjustment was repeated for the real measurement data acquired in the field. This way the reference estimate of all calibration parameters is obtained. Afterwards, the same adjustment was applied on the measurements from only the first scanner station S1 (Figure 8). This process failed as expected, what will be furtherly explained in the next section. Then, the selected subset of the calibration parameters sensitive to two-face measurements (Equations (17)–(19)) was estimated both using all three and only the first scanner station, for comparison purposes. Finally, the two-face calibration adjustment (Section 2.5) was applied on the measurements from the first scanner station. All of the estimated parameters together with corresponding precision are presented in the following section. The consistency of the estimated parameters with the reference ones is tested using the mentioned statistical approach. Additionally, the analysis of the parameter correlations was conducted. All of the relevant findings are described in Section 4.

## 4. Results and Discussion

In Section 4.1, the results of the simulation experiments are presented and discussed, while Section 4.2 deals with the results of the empirical experiment.

### 4.1. Simulation Results

Table 2 presents the results of the two self-calibration attempts based on the calibration algorithm described in Section 2.3 and Section 2.4, using the set of all simulated measurements from all three scanner stations. In this instance, the influence of the mechanical misalignments on the scanner measurements is simulated and, therefore, the true values of the calibration parameters are known (Section 3.2). Parameters *x*_5*z*−7_ and *x*_1*n*+2_ combine the influence of two misalignments and, therefore, their values are doubled. The first calibration attempt was conducted without introducing the additional calibration parameter *x*_1*n*+2_ in the vertical angle equation (Equation (6)). Instead, the parameters *x*_1*n*_ and *x*_2_ are estimated separately as: $+\frac{(x_{1n}+x_{2})\cos\left(\theta_{j}^{i}\right)}{r_{j}^{i}}$. As it can be seen from Table 2, this leads to a noticeable bias in the estimate of the parameters *x*_1*n*_ and *x*_5*n*_, which are highly correlated. In the second calibration attempt, the proposed parameter *x*_1*n*+2_ is introduced, and the estimate of the mentioned parameters is evidently improved. Therefore, only the second attempt is discussed in further detail.

Most of the parameters are estimated with an accuracy and precision of several tenths of arc seconds and several hundredths of millimetres. Two parameters (*x*_5*z*−7_ and *x*_5*n*_) are determined with a lower precision of approximately 2.5ʺ, but without bias, while two parameter estimates are somewhat biased (*x*_1*n*_ and *x*_5*z*_). The bias of the parameter *x*_1*n*_ estimate can be bypassed. Namely, the parameter *x*_1*n*+2_ contains the combined influence of the parameters *x*_1*n*_ and *x*_2_. Additionally, the parameters *x*_1*n*+2_ and *x*_2_ are estimated with the higher accuracy and precision than *x*_1*n*_. Hence, the better estimate of the parameter *x*_1*n*_ can be derived by subtracting the influence of the parameter *x*_2_ from *x*_1*n*+2_. The corresponding precision can also be estimated using the low of error propagation [6]. The value and the precision of the parameter *x*_1*n*_ estimated this way are: −0.25 and 0.05 mm. Therefore, the unbiased and more precise estimate is derived. For the evaluation, in one calibration attempt not provided herein, the parameter *x*_1*n*_ was completely removed from the calibration adjustment (from Equation (5)). This led to no noticeable differences in the calibration results. Hence, we kept the parameter for sake of comparison.

When analysing the overall maximum correlations, it is clear that most of the calibration parameters maintained very high correlations towards each other. This is expected because of the similar functional definition of some parameters. However, in this case, it seems that these correlations do not influence the parameter accuracy if the parameters are estimated with the sufficient precision. Therefore, these correlations will not be in the further focus of the work.

Problematic are high correlations with non-calibration parameters (EOPs and OPs), which lead to a bias in the parameter estimates. It is especially visible in the case of the parameter *x*_5*z*_, which is almost perfectly correlated with the translation parameter in the direction of the *z* axis of the second scanner station. These correlations should be mitigated with a better network design, and this is the part of ongoing investigation in a further study. Finally, most of the calibration parameters sensitive to two-face measurements (Equations (17)–(19)) have low correlations with EOPs and OPs.

The results of both calibration attempts were subjected to the congruency test (Equation (24)). As it can be seen in Table 3, the adjustment without introducing parameter *x*_1*n*+2_ clearly failed the statistical test. On the contrary, in the second case, the test statistic was lower than the threshold value, resulting in the test acceptance. This indicates that the network realized in the experiment is sensitive enough to estimate unbiased parameters if the proposed set of calibration parameters is used (Equations (4)–(6)). In other words, the estimated parameters do not significantly differ from their true values and the used network is proved to be valid for further analysis.

### 4.2. Empirical Results

Table 4 summarizes the results of the self-calibration adjustment using all measurements obtained on the field, from all three stations. In this instance, the true values of the calibration parameters are unknown and only the parameters validated in the previous section are used (Equations (4)–(6)). As it can be seen, the measurement precision and correlations do not noticeably differ from the simulation case. Most of the parameters are estimated with a precision noticeably lower than the measurement noise and would be determined significant by applying usual statistical tests based on the Fisher’s distribution (see [7]).

Table 5 presents the results of several different calibration attempts using only parameters sensitive to two-face measurements (Equations (17)–(19)). The first attempt (3 × SS) uses all of the measurements from all three scanner stations and it is only a reduced version of the calibration provided in Table 4. These are the reference results against which the calibration from a single scanner station is compared.

The number of parameters is reduced because the parameters *x*_10_ and *x*_5*z*_ cannot be estimated from a single scanner station without reference information, as explained in Section 2.3. Additionally, parameter *x*_1*n*_ needs to be estimated a posteriori as explained in the previous section (Section 4.1). We tried to directly estimate parameters *x*_10_, *x*_5*z*_ and *x*_1*n*_ from a single station in order to test these premises. While the inclusion of the parameters *x*_10_ and *x*_1*n*_ caused that the adjustment could not converge, the parameter *x*_5*z*_ was estimated. However, the estimated value of −97.97° was obviously false and the parameter was perfectly correlated with one point coordinate. Removing the mentioned three parameters leads to the successful calibration.

The following calibration attempts are realized using only the data from the first scanner station S1 (Figure 8). The second station S2 was not used due to the poor network geometry and the poor recoverability of the calibration parameters, while the third station S3 was not used due to the lack of two-face measurements. The self-calibration based on the usual calibration algorithm (Section 2.3 and Section 2.4) was conducted with two slightly different realizations. In the first attempt, a separate set of the exterior orientation parameters is used for each scan (1 × SS (2 × EOP)). This is the usual practice in the former works on this topic (e.g., [33]). However, the implicit formulation of the measurements in two faces (Section 2.1) justifies using only one set of exterior orientation parameters for both scans from the same scanner station (1 × SS (1 × EOP)). This newly proposed formulation of the adjustment will be proven to be mandatory for the TLS calibration from a single scanner station (Table 5). The last calibration attempt is realized using the proposed two-face adjustment.

As can be seen, all of the calibration attempts provided quite similar results. This intuitively shows that all of the selected calibration parameters can be estimated from a single scanner station. To justify this hypothesis, the congruency test (Equation (24)) was applied. Estimates of the complete network configuration with all stations (3 × SS) are compared with all three calibration attempts from the single scanner station. The results are presented in the Table 6.

In the case of the two-face adjustment and the adjustment with the one set of EOP parameters, the test statistics is lower than the threshold value and, therefore, the hypothesis is accepted. This means that estimates of these two approaches do not significantly differ from the full network calibration. However, the first case with the two sets of EOPs failed. The reason lies in the fact that the estimated EOPs have been misplaced and that caused the wrong parameter estimates. This means that the calibration from one scanner station significantly benefits from the inclusion of the implicit two-face measurements and reduction of the number of exterior orientation parameters from 12 to 6.

The reduction of the number of exterior orientation parameters in the case of the full network of several scanner stations was also investigated. The investigation suggested that changing the number of EOPs has no significant impact in this case. The most probable explanation for this is that the EOPs of each scan are very well controlled and precisely estimated in the network adjustment having more than one scanner station. In that case the adjustment does not benefit from this additional constraint—that two consecutive scans from the same scanner station (two-face measurements) share the same position and orientation.

From here, a conclusion can be made: two scans in the first and second cycle (Section 2.1) sharing the same set of EOPs are mandatory prerequisites for the TLS calibration from a single scanner station, if the scanner position is not well controlled. This control can be achieved by introducing the reference target coordinates retrieved with a more accurate instrument. In that case, the number of exterior orientation parameters used in the adjustment is irrelevant.

**Figure 9:**
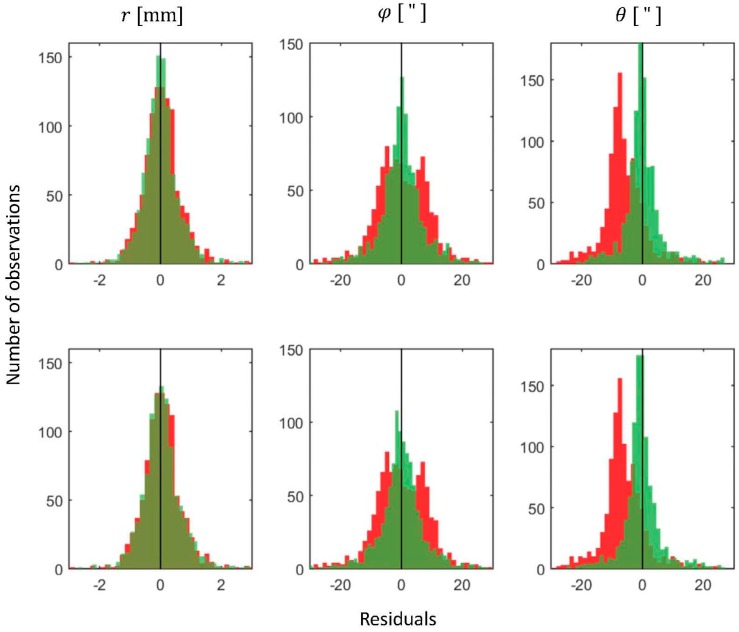
Histograms of the measurement residuals. The red color presents the residuals of the uncalibrated measurements, while the green color presents the residuals of the calibrated measurements (top: 3 × SS—calibration using parameters from Table 4, bottom: 1 × SS—calibration using parameters from the last two columns of Table 5).

Further on, we tested the improvement achieved using the estimated parameters. For this, the field measurements are corrected with two sets of the calibration parameters. The first parameter set is the reference estimate of all calibration parameters from Table 4, using all scanner stations, while the second set contains the parameters estimated from only one scanner station. For the latter one, the parameters estimated from the two-face adjustment were used (and they equal 1 × SS (1 × EOP)). These corrected measurements were used for the simultaneous registration of all scans, based on the algorithm described in Section 2.3 and Section 2.4, but without estimating the calibration parameters again. Figure 9 presents the histograms of the measurement residuals of both registrations against the histogram of the measurement residuals of the registration without any calibration.

As it can be seen, both sets of calibration parameters successfully reduced the visible systematic trends in the horizontal and vertical angle measurements, while the range measurements did not present any noticeable trend even in the reference data. The values of the estimated measurement precision ($\hat{\sigma }$) are presented in Table 7.

**Figure 10:**
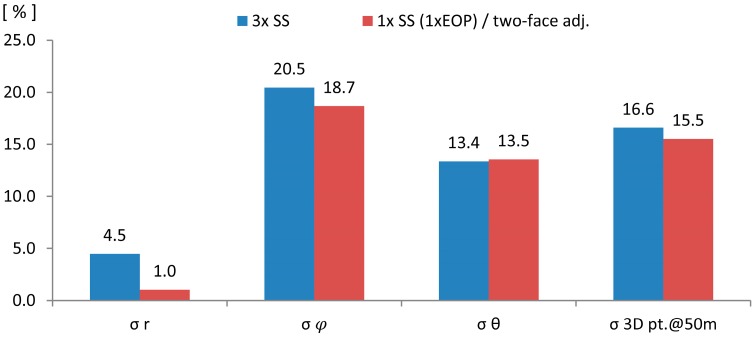
The improvement achieved by using the different sets of the calibration parameters in: estimated precision of ranges, horizontal and vertical angles and 3D point position at 50 m.

Again, the improvements achieved using the parameters estimated from all scanner stations do not noticeably differ from the improvements achieved using the parameters estimated from only one scanner station. Finally, the percentage of the improvements is depicted on Figure 10.

The highest difference between two realizations can be seen in the case of the range measurements. This is expected, because the most influential range parameter—rangefinder offset, was not estimated from a single scanner station. This can be solved by incorporating reference values in the adjustment, as explained in Section 2.2. The other improvements are comparable, indicating that the calibration from a single scanner station can indeed produce similar results to the self-calibration using the full network configuration.

In the end, one more thing should be highlighted: most of the previous calibration strategies suggested that two consecutive scans from a single scanner station should be rotated around the z axis by 60°, 90° or 120° in order to improve the estimates of the calibration parameters (e.g., [16,20,21,33]). However, from this experiment, another assumption could be derived. In the case of panoramic terrestrial laser scanners, the best influence on the parameter estimates was the inclusion of two consecutive scans rotated by 180° around the *z* axis.

## 5. Conclusions and Future Work

This study presents an approach for the system calibration of a panoramic terrestrial laser scanner for assuring high quality of laser scans. The three main aims of this paper were:

- To prove that most of the calibration parameters can be estimated from a single scanner station,
- To prove that the proposed two-face adjustment can yield similar results to more complex self-calibration based on the bundle adjustment and
- To present the adaptation of the mechanically interpretable calibration parameters to the system calibration of terrestrial laser scanners.

The validity of the proposed hypothesis was tested in the conducted experiments. The experiments took place in a large hall in order to improve the sensitivity of the calibration approach. A new parametrization based on the genuine scanner geometry was adapted to the self-calibration approach. Mechanical misalignments influencing the measurements were found in the scanner and the values of most of the calibration parameters were determined with notable precision. Additionally, the network design was tested and validated through a simulation process. Some conclusions can be drawn from this investigation:

- Most of the parameters can be estimated from a single scanner station, without need for any reference information. More precisely nine out of 11 calibration parameters were successfully determined this way. This means that calibration time can be considerably reduced, in the present case from approximately five to two hours.
- In order to estimate all mechanical parameters from a single scanner station, reference measurements are required. Namely, this is the case for the remaining two parameters that are not sensitive to two-face measurements (*x*_10_ and *x*_5*z*_).
- The proposed two-face adjustment can yield comparable results to the usual self-calibration strategies. Even though it is not rigorous, it is proven to be a fast and simple solution for the calibration from a single scanner station.
- The implementation of the new systematic error parametrization in the usual self-calibration approach requires some modifications. The most interesting one is the introduction of the calibration parameter *x*_1*n*+2_, which successfully eliminated the bias from some parameter estimates.
- Using the same set of exterior orientation parameters for two consecutive scans from a single scanner station (two-face measurements) is a mandatory prerequisite for the scanner calibration from only one station, if the position of the scanner is not adequately controlled.

For the future, we plan to repeat the experiment and employ a separate control in order to test the stability of the estimated parameters, which is the main motivation of using mechanical parameters. For the next calibration attempt, we will put significant effort in the realization of an improved network geometry in order to successfully detect and remove outliers, reduce remaining correlations and improve the sensitivity for estimating the remaining calibration parameters from a single scanner station. In the end, we will try to extend our solution on the parameter model which incorporates encoder related errors and a comprehensive rangefinder parametrization.

## Figures and Tables

**Table 1:** Comprehensive list of the mechanical calibration parameters [29] and their comparison with the parameters based on the total station model [21].

Parameter	Description	Equivalent in the Total Station Model
*x*_1*n*_	Horizontal beam offset	Horizontal eccentricity of collimation axis *
*x*_1*z*_	Vertical beam offset	None
*x*_2_	Horizontal axis offset	Laser axis vertical offset *
*x*_3_	Mirror offset	None
*x*_4_	Vertical index offset	Identical
*x*_5*n*_	Horizontal beam tilt	Vertical circle eccentricity error *
*x*_5*z*_	Vertical beam tilt	Horizontal axis & vertical circle eccentricity error
*x*_6_	Mirror tilt	Collimation axis error
*x*_7_	Horizontal axis error (tilt)	Identical
*x*_8*x*_	Horizontal angle encoder eccentricity	Identical
*x*_8*y*_	Horizontal angle encoder eccentricity	Identical
*x*_9*n*_	Vertical angle encoder eccentricity	Identical
*x*_9*z*_	Vertical angle encoder eccentricity	Identical
*x*_10_	Rangefinder offset	Identical
*x*_11*a*_	Second order scale error in the horizontal angle encoder	Identical
*x*_11*b*_	Second order scale error in the horizontal angle encoder	Identical
*x*_12*a*_	Second order scale error in the vertical angle encoder	Identical
*x*_12*b*_	Second order scale error in the vertical angle encoder	Identical

* Only partially modeled with the total station model.

**Table 2:** Comparison of the true and estimated values of the simulated calibration parameters. Correlations presented on the right hand side apply on the 2nd estimate, with first two columns indicating overall maximal correlations and last two columns indicating maximal correlations with respect towards exterior orientation (EOPs) and object point parameters (OPs). Notations: *XYZ*—N indicates correlation with one of the coordinates of the target N, while for example *Tx*_1_ and *Rx*_1_ indicate correlation with translation and rotation parameters with respect to *x* axis of the first scanner station.

Parameter	True	1st Estimate		2nd Estimate		Overall Corr.		w.r.t. EOPs & OPs	
	*x*	$\hat{x}$	${\hat{\sigma }}_{x}$	$\hat{x}$	${\hat{\sigma }}_{x}$	Corr.	With	Corr.	With
***x*_1*n*_ [mm]**	−0.20	0.07	0.02	−0.10	0.06	0.66	EOP-*Rz*_1_	0.66	EOP-*Rz*_1_
***x*_1*z*_ [mm]**	−0.20	−0.18	0.05	−0.18	0.05	−0.98	*x*_5*z*−7_	−0.62	EOP-*Tz*_3_
***x*_2_ [mm]**	−0.20	−0.18	0.02	−0.17	0.02	0.21	EOP-*Tx*_3_	0.21	EOP-*Tx*_3_
***x*_3_ [mm]**	−0.20	−0.20	0.02	−0.21	0.02	0.80	*x*_1*z*_	−0.49	EOP-*Tz*_3_
***x*_4_ ["]**	−8.00	−8.39	0.38	−7.97	0.30	−0.45	*x*_5*n*_	−0.21	EOP-*Ry*_3_
***x*_5*n*_ ["]**	−8.00	−15.52	1.54	−6.22	2.50	−0.86	*x*_1*n*+2_	0.11	*XYZ*-203
***x*_5*z*−7_ ["]**	−16.00	−17.83	2.22	−17.65	2.22	−0.98	*x*_1*z*_	0.60	EOP-*Tz*_3_
***x*_6_ ["]**	−8.00	−7.81	0.17	−7.79	0.17	−0.56	*x*_3_	−0.21	EOP-*Rz*_3_
***x*_5*z*_ ["]**	−8.00	−13.09	1.99	−12.55	1.98	−0.95	EOP-*Tz*_2_	−0.95	EOP-*Tz*_2_
***x*_10_ [mm]**	−2.00	−2.07	0.07	−2.06	0.07	−0.58	EOP-*Tx*_3_	−0.58	EOP-*Tx*_3_
***x*_1*n*+2_ [mm]**	−0.40	-	-	−0.42	0.05	−0.86	*x*_5*n*_	0.06	*XYZ*-209

**Table 3:** Congruency test (Equation (24)) for validation of the estimated parameters.

Estimate	$T_{c}$	$F_{\left(h,r,1-\alpha \right)}$
**1st estimate**	49.82	1.83
**2nd estimate**	1.63	1.79

**Table 4:** Estimated calibration parameters from the field experiment. The notation 3 × SS denotes that all three scanner stations were used in the calibration process.

All Parameters			Correlations			
Parameter	3 × SS		Overall Corr.		w.r.t. EOPs & OPs	
	$\hat{x}$	${\hat{\sigma }}_{x}$	Corr.	With	Corr.	With
***x*_1*n*_ [mm]**	−0.14	0.08	0.68	EOP-*Rz*_2_	0.68	EOP-*Rz*_2_
***x*_1*z*_ [mm]**	0.29	0.06	−0.98	*x*_5*z*−7_	−0.64	EOP-*Tz*_3_
***x*_2_ [mm]**	0.05	0.03	0.20	EOP-*Tx*_3_	0.20	EOP-*Tx*_3_
***x*_3_ [mm]**	−0.07	0.02	0.81	*x*_1*z*_	−0.52	EOP-*Tz*_3_
***x*_4_ ["]**	−6.83	0.34	−0.45	*x*_5*n*_	−0.21	EOP-*Ry*_3_
***x*_5*n*_ ["]**	−9.15	2.85	−0.86	*x*_1*n*+2_	−0.11	*XYZ*-208
***x*_5*z*−7_ ["]**	−19.78	2.69	−0.98	*x*_1*z*_	0.62	EOP-*Tz*_3_
***x*_6_ ["]**	3.64	0.21	−0.56	*x*_3_	−0.21	EOP-*Rz*_3_
***x*_5*z*_ ["]**	−5.11	2.34	−0.95	EOP-*Tz*_2_	−0.95	EOP-*Tz*_2_
***x*_10_ [mm]**	0.61	0.09	−0.59	EOP-*Tx*_3_	−0.59	EOP-*Tx*_3_
***x*_1*n*+2_ [mm]**	−0.17	0.05	−0.86	*x*_5*n*_	0.06	*XYZ*-209

**Table 5:** Comparison of the different calibration attempts estimating only two-face sensitive calibration parameters (Equations (17)–(19)). Notation explanation: 3 × SS—calibration using measurements from all three scanner stations, 1 × SS (2 × EOP)—calibration using only data from the first scanner station and assigning different exterior orientation parameters for each scan, 1 × SS (1 × EOP)—assigning one set of exterior orientation parameters for both scans, 1 × SS (two-face adj.)—calibration using the two-face adjustment algorithm.

Two-Face Sensitive Parameters								
Parameter	3 × SS		1 × SS (2 × EOP)		1 × SS (1 × EOP)		1 × SS (Two-Face Adj.)	
	$\hat{x}$	${\hat{\sigma }}_{x}$	$\hat{x}$	${\hat{\sigma }}_{x}$	$\hat{x}$	${\hat{\sigma }}_{x}$	$\hat{x}$	${\hat{\sigma }}_{x}$
***x*_1*n*+2_ [mm]**	−0.18	0.05	−0.33	0.08	−0.28	0.08	−0.28	0.10
***x*_1*z*_ [mm]**	0.27	0.06	0.20	0.09	0.33	0.09	0.33	0.11
***x*_2_ [mm]**	0.06	0.03	−0.01	0.04	0.06	0.04	0.06	0.02
***x*_3_ [mm]**	−0.08	0.02	−0.15	0.04	−0.13	0.03	−0.13	0.04
***x*_4_ ["]**	−6.86	0.35	−5.00	0.75	−6.75	0.51	−6.75	0.61
***x*_5*n*_ ["]**	−8.54	2.88	−4.37	4.03	−4.21	4.07	−4.21	4.88
***x*_5*z*−7_ ["]**	−18.83	2.71	−16.28	3.92	−20.99	3.71	−20.99	4.59
***x*_6_ ["]**	3.65	0.21	4.51	0.36	4.50	0.33	4.50	0.41

**Table 6:** Congruency test used for evaluating if the parameter estimates from only one scanner station are significantly different from the calibration using all scanner stations (rejection signalizing the significant difference).

Calib. Attempts	$T_{c}$	$F_{\left(h,r,1-\alpha \right)}$
**1 × SS (2 × EOP)**	2.26	1.94
**1 × SS (1 × EOP)**	1.27	1.94
**1 × SS (two-face adj.)**	1.23	1.94

**Table 7:** The estimated precision for the: ranges, horizontal and vertical angles and 3D point position at 50 m (first row—uncalibrated measurements, 3 × SS—measurement calibrated with the parameters from Table 4, 1 × SS—measurements calibrated with the parameters from last two columns of Table 5).

$\hat{\sigma }$	r [mm]	φ ["]	θ ["]	3D pt.@50 m [mm]
**Without calibration**	0.78	10.03	8.61	3.30
**3 × SS**	0.75	7.98	7.46	2.75
**1 × SS**	0.78	8.16	7.45	2.79
